# Editorial: Adipose Tissue: Which Role in Aging and Longevity?

**DOI:** 10.3389/fendo.2020.00583

**Published:** 2020-08-25

**Authors:** Antonello Lorenzini, Daniela Monti, Aurelia Santoro

**Affiliations:** ^1^Biochemistry Unit, Department of Biomedical and Neuromotor Sciences, University of Bologna, Bologna, Italy; ^2^Department of Experimental and Clinical Biomedical Sciences “Mario Serio”, University of Florence, Florence, Italy; ^3^Department of Experimental, Diagnostic and Specialty Medicine, Alma Mater Studiorum, University of Bologna, Bologna, Italy

**Keywords:** body fat, adipose tissue, obesity, adipokines, adiponectin, aging, longevity

Since 2018, we are living in a world where there are more people over age 65 than there are children under five. Predictions indicate, if this trend continues, by the year 2050, the number of people over 65 will be double the number of people under five (1). Consequently, an understanding of the optimal physiological, endocrinological, and anthropometric conditions associated with better health during aging is to be considered a priority topic. In parallel with the increasing aging of the population, there is a parallel increase of overweight and obese individuals among older adults (2).

Normal aging involves important changes to body composition, including decreased muscle mass and increased fat mass (3). Basal metabolism, for the majority of the elderly, is the main daily energetic expenditure and its decrease with age provides one explanation for the tendency to gain weight, with age. In addition to this physiological statement, lifestyle changes in aged people and the associated reduction in physical activity level favors weight increase with age. Total body fat peaks at about 65–70 years, while in advanced old age it decreases. Aging, indeed, modifies adipose tissue accumulation and redistribution resulting in accumulation of abdominal fat. These age-related changes alter many physiological functions including inflammation and contribute to age-related diseases such as cardiovascular events, diabetes mellitus, hypertension, stroke, and several types of cancer (4). However, to what extent, the age-related adipose tissue remodeling impacts the health status in elderly is incompletely understood.

To highlight and clarify the main age-related changes in adipose tissue and discuss its implications on health status with particular regard to age-related diseases, we dedicated a Research Topic to the alteration of lipid storage, the redistribution and the types of fat, the production of different mediators contributing to a pro-inflammatory status in aging.

Conte et al. are setting the stage, discussing the evident evolutionarily advantage provided by this tissue common among all animal species. Maintaining the correct distribution of body fat seems crucial for health and longevity. Interestingly, it seems that while a lower threshold of fat mass exists, it does not appear existing an upper one. In human and in many animals, adipose tissue can be accumulated in very large amounts. Most probably, an upper limit was not established by natural selection because a large accumulation of body fat in the wild is uncommon, unlike what we are observing during modern times in our species. Although the health implication of excessive body fat is evident, as they discuss, they also propose that a suitable amount of fat is probably an important feature for reaching extended longevity (Conte et al.).

Because of its simplicity, BMI is broadly used as a surrogate for body fat, although it is highly imprecise. For example, a bodybuilder with a low percentage of body fat could fall in the obese category. Ponti et al. present how body composition is different at different ages, stressing that there is not only an increase in body fat but also a redistribution of body mass with age. In particular, fat mainly increases in the trunk (largely visceral fat), but not in arms or legs. A major difference also exists between male and female older adults likely contributing to the sex-difference in the prevalence of age-related diseases. An accurate assessment of body composition is critical to discriminate an increase/decrease of fat rather than muscle mass in the elderly. Ponti et al., review the most precise methods available for the clinic and for research to determine body composition [dual-energy X-ray absorptiometry (DXA), ultrasound, computed tomography (CT) and magnetic resonance imaging (MRI)] outlining advantages and disadvantages of each technique.

Zoico et al. focus on the significance of changes happening during aging in two subcategories of body fat: brown adipose tissue (BAT) and beige adipose tissue, fat tissues rich in mitochondria with the univocal (brown) or conditional (beige) function of converting stored energy into heat.

Adipose tissue is a recognized endocrine organ, producing a variety of adipokines, whose levels tend to increase with aging. Mancuso and Bouchard have provided a comprehensive overview of adipokine functions, classifying them as pro-inflammatory (leptin, resistin, chemerin, retinol binding protein 4, lipocalin 2, CCL2, IL-1β, IL-6, IL-12, IL-18, and TNF-α) and anti-inflammatory (adiponectin, vaspin, secreted-frizzled-related protein 5, omentin-1 C1q/TNF-related proteins).

Arai et al. focus on the roles and significance of adiponectin, an adipokine whose levels are elevated in centenarians. In contrast to the majority of other adipokines, its plasma levels are inversely related to body fat. In this report, the authors describe how this adipokine is considered highly beneficial for longevity, possibly contributing to enhancing insulin sensitivity. They also describe some interesting paradoxes related to adiponectin that challenge its beneficial role: the observed association between higher adiponectin level and mortality in patients with cardiovascular disease and with frailty in elderly subjects. They propose a solution to these paradoxes introducing the concept of adiponectin resistance: higher adiponectin levels, in their view, is possibly a compensatory mechanism in response to inflammation and oxidative stress.

In light of the current SARS-CoV-2 pandemic affecting prevalently the elderly (5), an important topic is the role of the process of aging in the susceptibility to infectious diseases. Obesity, as it increases with age, exerts a cumulative effect. Obese individuals are increasingly vulnerable to fungal, bacterial, and viral infection. Frasca and McElhaney present an overview of the roles of obesity on the immune response to respiratory tract infection. Specifically, they analyze the risk for the elderly represented by pneumococcus infection, highlighting the presence of an interesting obesity paradox: it appears that obesity is protective against the more serious complications of this bacterial infection. This stresses the need to investigate further, how obesity is modulating our immune response (Frasca and McElhaney).

Salvestrini et al. look from further away at the interrelationship between excess body fat and aging. Their considerations stem from a reflection on the experimental paradigm of life span extension by caloric restriction, specifically on how best to consider control animals when translating experimental results to human (6). If a control animal, *ad libitum* fed, has to be considered an animal with no excess fat, equivalent to a normal weight human (BMI between 18.5 and 24.9) than to benefit from the lifespan-extending effect of CR, a human should approach underweight. If, instead, as many authors are proposing [reviewed in (6)], control animals in many instances should be considered the equivalent of obese humans, then the lifespan-extending capacity of CR is simply communicating that obesity has a life shortening effect, which is well-known from epidemiological evidence. From these considerations Salvestrini et al. have looked at obesity under the lens of the hallmarks of aging as listed by López-Otín et al. (7); the vast collection of literature they overviewed, demonstrates an impressive overlap between the process of aging and the metabolic consequences of excess body fat (Salvestrini et al.).

Although the increase of body fat with age remains a major risk factor for age-related diseases, several studies are needed to disentangle the complex network of metabolic, endocrinological, and immunological mediators that are involved. Moreover, the general increase of the elderly population leads to the consequent increase of 90+ elderly and centenarians. Many studies demonstrated the peculiarity of these individuals (8, 9), however little is known about the amount and kind of adipose tissue they have. Future researches are needed to investigate the age-related remodeling of body fat including also very old people.

## Author Contributions

AL wrote the initial draft. AS and DM implemented and revised it. All authors gave final approval of the submitted version.

## Conflict of Interest

The authors declare that the research was conducted in the absence of any commercial or financial relationships that could be construed as a potential conflict of interest.
